# Increased Mesenchymal Stem Cell Response and Decreased *Staphylococcus aureus* Adhesion on Titania Nanotubes without Pharmaceuticals

**DOI:** 10.1155/2015/172898

**Published:** 2015-11-10

**Authors:** Zhiqiang Xu, Yingzhen Lai, Dong Wu, Wenxiu Huang, Sijia Huang, Lin Zhou, Jiang Chen

**Affiliations:** ^1^Department of Stomatology, Affiliated Hospital of Putian University, Putian, Fujian 351100, China; ^2^School of Stomatology, Fujian Medical University, Fuzhou, Fujian 350000, China; ^3^Department of Oral Medicine, Xiamen Medical College, Xiamen, Fujian 361000, China; ^4^Department of Oral Implantology, Affiliated Stomatological Hospital of Fujian Medical University, Fuzhou, Fujian 350002, China

## Abstract

Titanium (Ti) implants with enhanced biocompatibility and antibacterial property are highly desirable and characterized by improved success rates. In this study, titania nanotubes (TNTs) with various tube diameters were fabricated on Ti surfaces through electrochemical anodization at 10, 30, and 60 V (denoted as NT10, NT30, and NT60, resp.). Ti was also investigated and used as a control. NT10 with a diameter of 30 nm could promote the adhesion and proliferation of bone marrow mesenchymal stem cells (BMSCs) without noticeable differentiation. NT30 with a diameter of 100 nm could support the adhesion and proliferation of BMSCs and induce osteogenesis. NT60 with a diameter of 200 nm demonstrated the best ability to promote cell spreading and osteogenic differentiation; however, it clearly impaired cell adhesion and proliferation. As the tube diameter increased, bacterial adhesion on the TNTs decreased and reached the lowest value on NT60. Therefore, NT30 without pharmaceuticals could be used to increase mesenchymal stem cell response and decrease *Staphylococcus aureus* adhesion and thus should be further studied for improving the efficacy of Ti-based orthopedic implants.

## 1. Introduction

Titanium (Ti) implants are widely used clinically because of their high biocompatibility and good mechanical properties [1]. However, implant failure still occurs because of poor osseointegration and bacterial infection [2–4]. Bioactive molecules have also been introduced into Ti implant surface to improve bone cell functionalities [5, 6], but these coatings are unstable and fabrication is typically time consuming and costly. Antibiotics are commonly administered to treat medical-device-related infections; nevertheless, the efficiencies of antibiotics have decreased because of the increased occurrence of multiple antibiotic-resistant bacterial strains [3, 7]. Hence, implants with enhanced osteogenic activity and antibacterial property without pharmaceuticals should be developed.

Since the bone itself has a nanoscale hierarchical structure, titania nanotubes (TNTs) fabricated on the Ti surface by anodization have received considerable attention in orthopedic research [8–13]. Small (25–30 nm diameter) anatase phase nanotubes enhance the adhesion of bone mesenchymal stem cells (BMSCs), while large (70–100 nm diameter) anatase phase nanotubes improve the osteogenic differentiation of BMSCs, without pharmaceutical agents [11, 13]. Cell proliferation on the large nanotubes, which is inhibited in the early stages because of the reciprocal relationship between cell proliferation and differentiation, catches up due to the larger surface area available for cell colonization after prolonged incubation [13]. TNTs with a diameter of >100 nm are difficult to fabricate using electrolytes containing 0.5% HF; however, nanotubes with a diameter of 100 nm are not the threshold influencing cell behaviors [11].

Similar to BMSCs, nanoscale patterning can influence bacterial adhesion [14]. TNTs decrease bacterial adhesion even without pharmaceutical agents, which may elicit harmful side effects [15–18]. However, TNTs alone also have been reported to increase bacterial adhesion [18, 19]. These inconsistent results can be due to the disparity in nanoscale features and physical properties. Though numerous studies have focused on the effects of TNTs on the response of bacteria [15–19], few studies have simultaneously investigated the effects of TNTs on differentially modulating BMSCs and bacterial adhesion. Thus, the same TNTs substrate for both BMSCs and bacteria should be evaluated when conflicting antibacterial assay results are considered.

This work sought to develop appropriate TNTs for implants to improve BMSCs osteogenic differentiation without blocking cell proliferation and simultaneously decrease* Staphylococcus aureus* adhesion without pharmaceuticals. TNTs with three different diameters (30, 100, and 200 nm) were prepared by using a weakly acidic electrolyte containing NH_4_F and glycerol. For the first time, the response of BMSCs and the adhesion of* S. aureus* on the three TNTs were systematically investigated simultaneously.

## 2. Materials and Methods

### 2.1. Fabrication of TNTs on Ti

TNTs were fabricated through anodization on a Ti sheet. Ti samples (1 cm × 1 cm × 0.025 cm, 99.8% purity; Alfa-Aesar, Ward Hill, MA, USA) were ultrasonically cleaned with acetone and deionized water for 15 min. The samples were eroded in 4 wt% HF-5 mol/L HNO_3_, rinsed with distilled water, and dried in air. Ti was used as the working electrode and a platinum sheet was used as the cathode. Anodization was performed using a mixture of 0.50 wt% NH_4_F + 10 vol% H_2_O in glycerol at 10, 30, and 60 V for 5 h, respectively, and designated as NT10, NT30, and NT60, respectively. After anodization, the samples were rinsed with deionized water, dried in air, and annealed at 450°C for 2 h to transform the as-anodized amorphous TNTs into the crystalline phase. An identically sized Ti sample with a native TiO_2_ oxide layer was used as the control. Both sides of the samples were sterilized by ultraviolet irradiation before use.

### 2.2. Specimen Characterization

#### 2.2.1. Scanning Electron Microscopy (SEM)

SEM (JSM-7500F; JEOL, Tokyo, Japan) was employed to evaluate the surface morphology. The samples were dried and sputter-coated with platinum prior to SEM examination.

#### 2.2.2. Phase Analysis

Phase analysis was conducted through X-ray diffraction (XRD), which was performed on X'Pert MPD (Philips, AMS, Netherlands) using Cu Ka radiation (*λ* = 0.15405 nm) with a scan rate of 0.02° and 0.3° per minute.

#### 2.2.3. Contact Angle Determination

About 1 *μ*L distilled water was dropped from the tip of a microliter syringe to the surface of the samples. Contact angles were measured on the obtained photographs (Phoenix 300; SEO, Seoul, Korea).

#### 2.2.4. Protein Adsorption Assay

A 1 mL droplet of Dulbecco's Modified Eagle's Medium-Low Glucose (DMEM-LG, Hyclone, USA) containing 10% fetal bovine serum (FBS, Hyclone, USA) was pipetted onto each sample. After incubation in the medium at 37°C for 2 h, the samples were transferred to new 24-well plates and gently rinsed three times with phosphate-buffered saline (PBS, Hyclone, USA), and then the proteins adsorbed onto the samples were detached by 1% sodium dodecyl sulfate and determined using a MicroBCA protein assay kit (Pierce, Rockford, IL, USA).

### 2.3. Cell Cultures

Sprague-Dawley rat BMSCs were purchased from Cyagen Biosciences (Guangzhou, China). The cells were routinely cultured according to the instructions of the supplier. Cells were used between passage 4 and passage 6 in the following experiments. The samples were placed in 24-well plates, and the BMSCs were seeded at a density of 4 × 10^4^/well for the cell adhesion assay and 2 × 10^4^/well for the other assays.

#### 2.3.1. Cell Morphology

After culturing for 2 d, the samples were rinsed with PBS, fixed with 3% glutaraldehyde, and dehydrated in graded ethanol series. Prior to SEM observation, the specimens were freeze-dried and sputter-coated with platinum layers.

#### 2.3.2. Adhesion and Proliferation

At the prescribed time points, the samples were transferred to new 24-well plates and gently rinsed three times with PBS. For the cell adhesion assay, after culturing for 0.5, 1, and 2 h, the attached cells on the samples were fixed, stained with 4,6-diamidino-2-phenylindole (DAPI; Sigma-Aldrich), and counted from five random fields on each sample using a fluorescence microscope. For the cell proliferation assay, the cells were cultured on the samples for 1, 3, and 7 days and the cell numbers were assessed by using Cell Counting Kit-8 (CCK-8; Beyotime, Shanghai, China) assay.

#### 2.3.3. Gene Expressions

The expression levels of osteogenesis related genes, including runt-related transcription factor 2 (RUNX2, a key transcript factor for osteogenic differentiation), alkaline phosphatase (ALP, an early marker for osteogenic differentiation), osteocalcin (OCN, a late marker for osteogenic differentiation), and type 1 collagen (COL-1, a main collagen found in bones), were measured using quantitative reverse transcription polymerase chain reaction (qRT-PCR). Total RNA was extracted using Trizol (Invitrogen, Carlsbad, CA, USA) after culturing for 2 weeks. An equivalent amount of RNA from each sample was then reverse transcribed with a cDNA Reverse Transcription Kit (TaKaRa, Shiga, Japan). The qRT-PCR analysis was performed on an ABI Prism 7500 real-time PCR cycler (Applied Biosystems, Carlsbad, CA, USA) using SYBR Premix Ex Taq II (TaKaRa). The primer sequences of the genes are shown in Table 1. The expression levels of the target genes were normalized to that of the housekeeping gene GAPDH.

### 2.4. Antibacterial Assay

*S. aureus* (ATCC25923; American Type Culture Collection, Manassas, VA, USA) was cultivated in the brain-heart infusion broth medium at 37°C for 12 h and resuspended at a concentration of 10^6^ CFU/mL. The samples were separately incubated in 1 mL of the bacteria-containing medium on 24-well culture plates.* In vitro* antibacterial activity was assessed by the plate-counting method. After culturing for 6 h, the sample was rinsed with PBS and ultrasonically agitated to detach the bacteria from the sample. The viable bacteria in the PBS were quantified by plating serial dilutions on agar plates. The agar plates were incubated at 37°C and the colony forming units (CFU) were counted visually after 2 h.

### 2.5. Statistical Analysis

All data were expressed as the mean ± standard deviation (SD). One-way ANOVA and Student-Newman-Keuls* post hoc* test were used to determine the level of significance. *p* < 0.05 was considered significant, and *p* < 0.01 was considered highly significant.

## 3. Results

### 3.1. Specimen Characterization

#### 3.1.1. SEM

**Figure 1:**
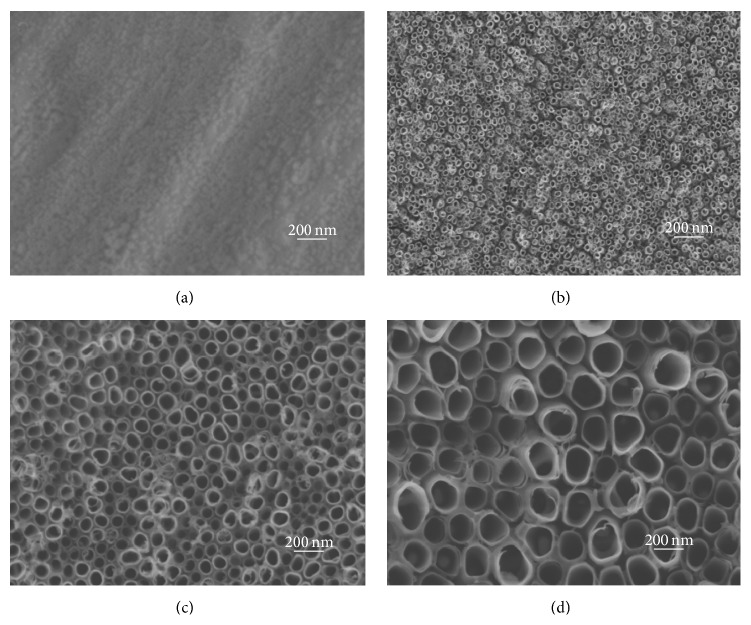
SEM images: (a) pure Ti, (b) NT10 with a diameter of 30 nm, (c) NT30 with a diameter of 100 nm, and (d) NT60 with a diameter of 200 nm.

SEM images show the surface topographies of the samples in Figure 1. Pure Ti possessed micron rough surface features and lacked obvious nanocues; by contrast, NT10, NT30, and NT60 had highly ordered nanotubes with diameters of about 30, 100, and 200 nm, respectively.

#### 3.1.2. XRD

**Figure 2:**
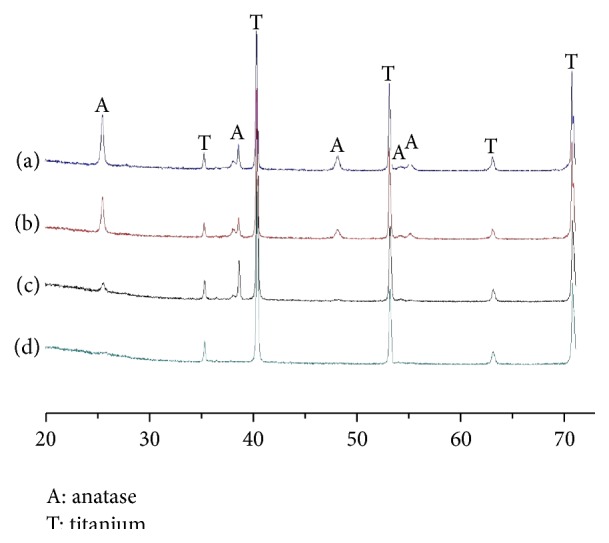
XRD patterns of TNTs after heat treatment at 450°C for 2 h: (a) NT10, (b) NT30, (c) NT60, and XRD pattern of (d) NT10 before heat treatment.

XRD results (Figure 2) indicated that, after annealing at 450°C for 2 h, all three types of nanotubes had Ti and anatase peaks but did not show any amorphous or rutile peaks. As a control, NT10 only had Ti peaks before annealing and did not show any anatase or rutile peaks.

#### 3.1.3. Static Contact Angles

**Figure 3:**
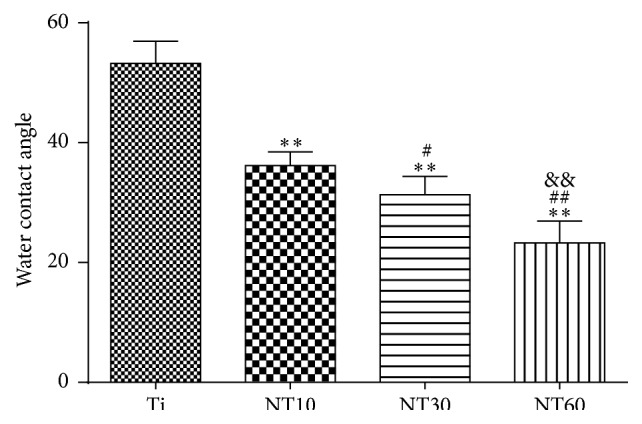
Multiple-comparison results of contact angles on different samples (mean ± SD, *N* = 5, and ^*∗*^ *p* < 0.05 and ^*∗∗*^ *p* < 0.01 compared with the Ti; ^#^ *p* < 0.05 and ^##^ *p* < 0.01 compared with the NT10; ^&^ *p* < 0.05 and ^&&^ *p* < 0.01 compared with the NT30).

The wettability of the separate surfaces was determined by measuring water contact angles (Figure 3). The smaller the contact angle, the greater the hydrophilicity. TNTs showed that surface hydrophilicity properties increased as the diameter increased. By contrast, pure Ti was fairly hydrophobic.

#### 3.1.4. Protein Adsorption Assay

**Figure 4:**
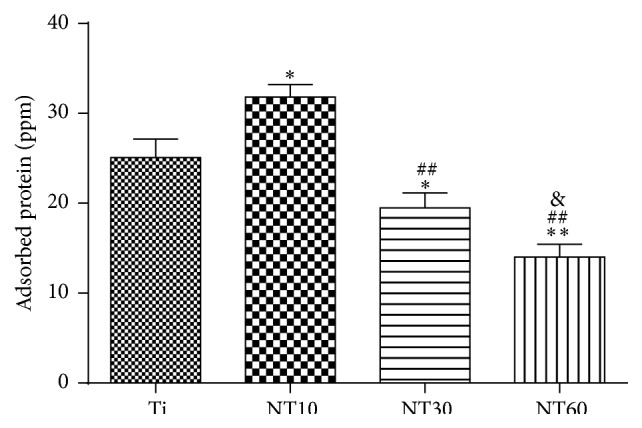
Protein adsorption to the samples after 2 h of immersion in DMEM–LG containing 10% FBS (mean ± SD, *N* = 3, and ^*∗*^ *p* < 0.05 and ^*∗∗*^ *p* < 0.01 compared with the Ti; ^#^ *p* < 0.05 and ^##^ *p* < 0.01 compared with the NT10; ^&^ *p* < 0.05 and ^&&^ *p* < 0.01 compared with the NT30).

The amounts of adsorbed proteins from 10% FBS after 2 h of incubation are shown in Figure 4 to elucidate subsequent cellular responses. NT10 absorbed more proteins than Ti; by contrast NT30 and NT60 absorbed fewer proteins than Ti.

### 3.2. *In Vitro* Biocompatibility Studies

#### 3.2.1. Cell Morphology

**Figure 5:**
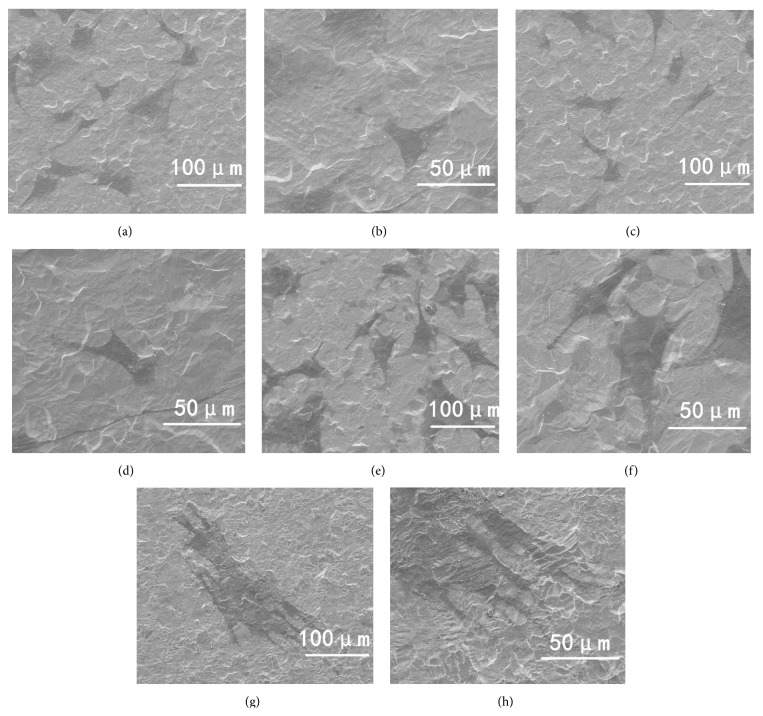
SEM images at 800x and 2,000x showing the morphological characteristics of BMSCs after 2 d of culture on the samples.

The shapes of the BMSCs cultured on the different surfaces were noticeably different, as shown in Figure 5. Most of the BMSCs on control Ti and NT10 had more rounded shapes without the noticeable filopodia extensions and cellular propagation (Figures 5(a)–5(d)). By contrast, the cells on NT30 and NT60 (Figures 5(e)–5(h)) became increasingly elongated with increased diameter and showed a large number of prominent filopodia and unidirectional lamellipodia extensions as the diameter increased.

#### 3.2.2. Adhesion and Proliferation

**Figure 6:**
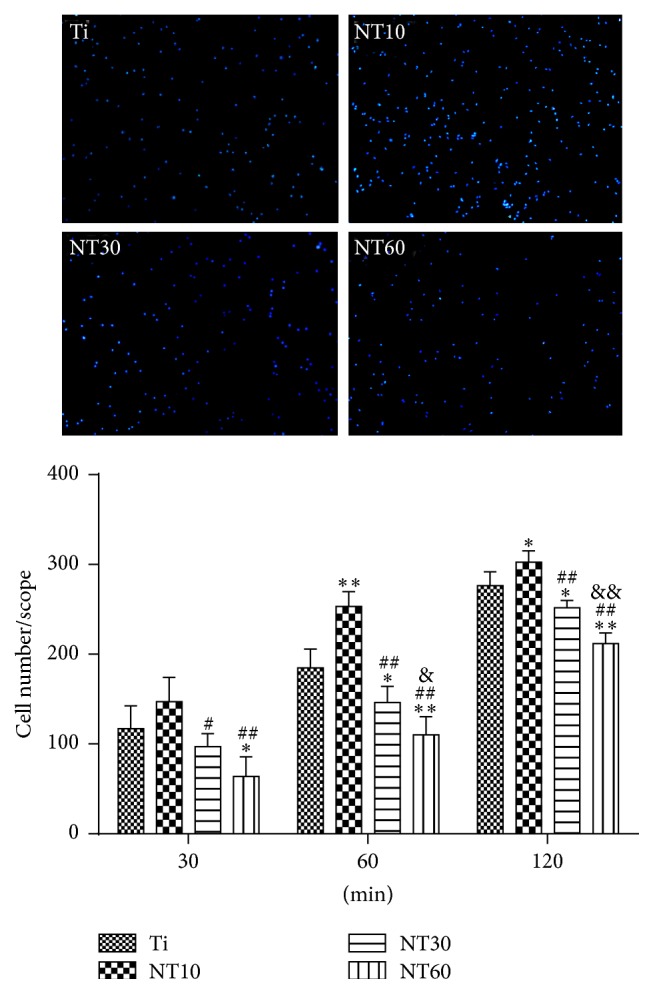
Fluorescence images of initial adherent BMSCs stained with DAPI after 1 h and cell numbers measured by counting cells for 0.5, 1, and 2 h (mean ± SD, *N* = 3, and ^*∗*^ *p* < 0.05 and ^*∗∗*^ *p* < 0.01 compared with the Ti; ^#^ *p* < 0.05 and ^##^ *p* < 0.01 compared with the NT10; ^&^ *p* < 0.05 and ^&&^ *p* < 0.01 compared with the NT30).

**Figure 7:**
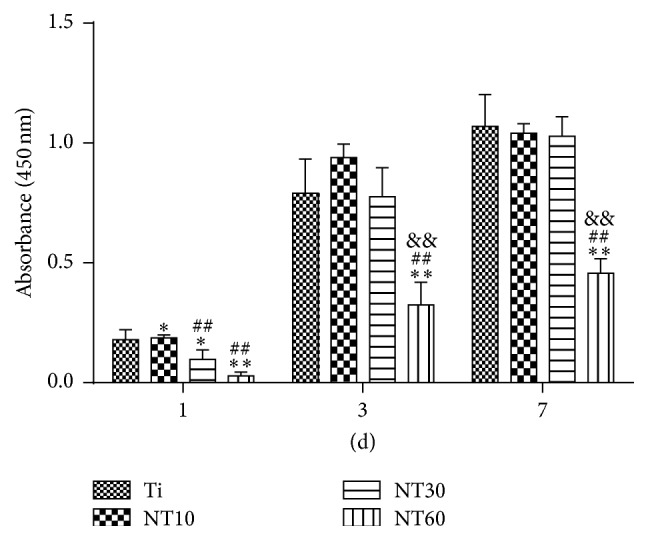
Cell proliferation measured by CCK-8 assay after culturing BMSCs on three different samples for 1, 3, and 7 d (mean ± SD, *N* = 3, and ^*∗*^ *p* < 0.05 and ^*∗∗*^ *p* < 0.01 compared with the Ti; ^#^ *p* < 0.05 and ^##^ *p* < 0.01 compared with the NT10; ^&^ *p* < 0.05 and ^&&^ *p* < 0.01 compared with the NT30).

The initial adherent cell number was measured by DAPI staining, as shown in Figure 6. Cell attachment on NT10 was significantly improved compared with control Ti surfaces for 1 h and 2 h. In contrast, cell attachment was inhibited on NT30 and NT60 at each time interval adopted in this study. Cell proliferation was measured by the CCK-8 assay (Figure 7). NT10 could promote cell proliferation compared with control Ti on day 1, but there was no significant difference by day 3 and day 7. Though on day 1 the cell numbers on NT30 were slightly lower than those on Ti or NT10, no obvious difference in the cell numbers was observed after culturing for 3 and 7 d. However, cell proliferation was severely inhibited on NT60 at any time.

#### 3.2.3. Gene Expressions

**Figure 8:**
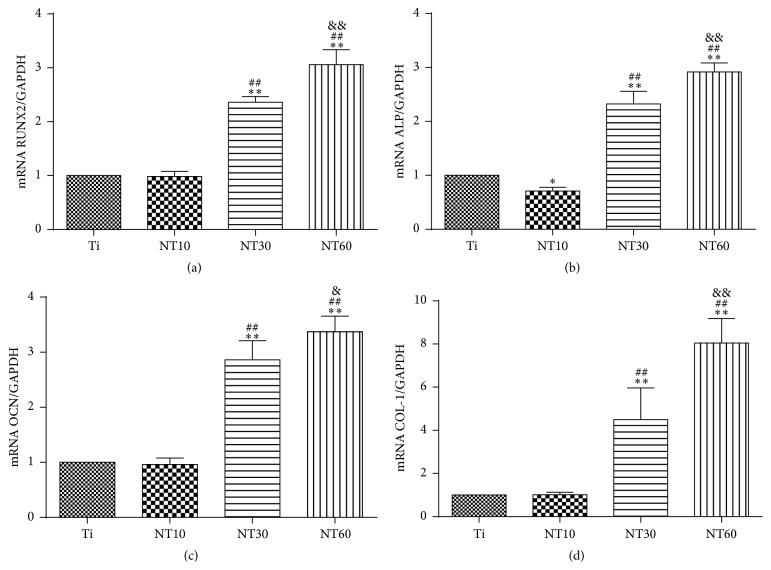
Relative expressions of (a) RUNX2, (b) ALP, (c) OCN, and (d) COL-1 by BMSCs seeding on different substrates for 2 weeks (mean ± SD, *N* = 3, and ^*∗*^ *p* < 0.05 and ^*∗∗*^ *p* < 0.01 compared with the Ti; ^#^ *p* < 0.05 and ^##^ *p* < 0.01 compared with the NT10; ^&^ *p* < 0.05 and ^&&^ *p* < 0.01 compared with the NT30).

The expression levels of osteogenesis related genes including RUNX2, ALP, OCN, and COL-1 were assessed by qRT-PCR (Figure 8). Ti and NT10 exhibited comparable expression levels, although the expression of ALP on the NT10 surface was slightly lower than that on the Ti surface. By contrast, the expression levels of osteogenesis related genes became increasingly higher as the diameter of NT30 and NT60 increased.

### 3.3. Antibacterial Activity

**Figure 9:**
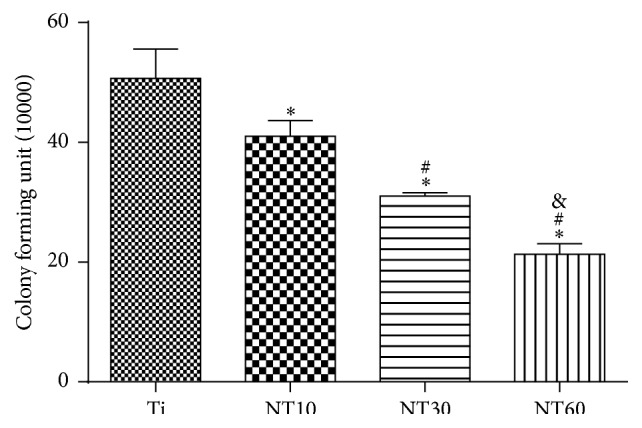
The quantitative analysis of viable adherent* S. aureus* on three different samples after 6 h (mean ± SD, *N* = 3, and ^*∗*^ *p* < 0.05 and ^*∗∗*^ *p* < 0.01 compared with the Ti; ^#^ *p* < 0.05 and ^##^ *p* < 0.01 compared with the NT10; ^&^ *p* < 0.05 and ^&&^ *p* < 0.01 compared with the NT30).

A quantitative spread plate method was adopted to evaluate viable bacteria on different samples after 6 h, as shown in Figure 9. The number of viable bacteria on the TNTs was significantly lower than that on the Ti; the number of viable bacteria decreased as the diameter of TNTs increased.

## 4. Discussions

Implants with both enhanced biointegration and antibacterial properties but without pharmaceutical agents should be developed. In the present study, TNTs with three various diameters were fabricated using weakly acidic electrolyte including NH_4_F and glycerol, which can generate larger and smoother nanotubular layers than using strong acid electrolyte containing HF [20]. Though NT60 exhibited the best ability to promote BMSCs osteogenic differentiation and prevent* S. aureus* adhesion, it obviously impaired cell adhesion and proliferation. NT10 could promote cell adhesion and proliferation but without noticeable differentiation. NT30 not only supported BMSCs adhesion and proliferation but also showed better antibacterial and osteogenesis-inducing ability; thus, NT30 has promising application in orthopedic implants.

A basic requirement for the use of biomaterials in bone is that they are biocompatible to bone cells, particularly to cells of the osteoblast lineage. BMSCs are the first cells to colonize the biomaterial surface after implantation, and most osteoblastic cells that colonize the implant surface to induce bone growth originate from BMSCs [21]. Hence, the behavior of BMSCs on TNTs should be evaluated before clinical application. The absorbed proteins convey the effect of topographical cues to the attached cells/tissues and direct the biological performance of biomaterials [4, 11, 21]. Cell attachment is significantly stronger on hydrophilic surfaces than on hydrophobic surfaces because of greater protein adhesion [22–26]. Thus, NT10 (30 nm in diameter) with high hydrophilicity induced more cell attachment at an early time than Ti because of greater protein adhesion. However, NT30 (100 nm in diameter) and NT60 (200 nm in diameter) with higher hydrophilicity had fewer protein aggregates and thus induced less cell attachment, with the latter the least. It is because the protein aggregates (≈30 nm-size regime) initially attach only to the available surfaces that are the top portion of the nanotube walls [11, 12], and these aggregates are too small to anchor on NT30, especially on NT60.

BMSCs cultured on TNTs had to extend across the tubes to find a protein-deposited surface for initial contact, thereby expanding their filopodia further and forming more extraordinarily elongated shapes. As the nanotube diameter increased from 30 nm to 200 nm, we found a clear trend of increasing cell elongation. Previous studies reported that elongated cell shapes are prone to undergo osteogenesis [27, 28]. Therefore NT30 and NT60 significantly promoted the expression of osteogenesis related genes and demonstrated excellent osteogenic activity, with the latter exhibiting a higher promotion. BMSCs on NT10 had a rounded shape without noticeable cellular extension and filopodia propagation, thus without noticeable osteogenic differentiation.

To estimate the density of viable cells, the cell proliferation assay was employed. Though NT10 could promote cell proliferation compared with control Ti on days 1 and 3, there was no significant difference by day 7 because of cell conjugation. Cell proliferation on NT30 was lower than that on the Ti control because of the reciprocal relationship between cell proliferation and differentiation [29, 30]. However, the inhibitory effect was neither serious nor long. By days 3 and 7, the cell growth on NT30 caught up because of the large surface area available for cell colonization and increased fluid exchange. This finding indicated that NT30 did not impair cell viability and could support cell proliferation. However, proliferation was severely impaired on NT60 and this trend became more evident with time. The extremely low adhesion at the early stage can potentially lead to cell quiescence or even apoptosis by anoikis, a type of programmed cell death through “homelessness” [31].

Bacteria adhesion plays an important role in the success rate of an implant. Since it is hard to kill bacteria after binding to the implant surface through antibiotic therapy, removal of the contaminated implant is often the only way to treat infections [32, 33]. Hence, new Ti-based biomaterials with desirable antibacterial properties are essential to prevent bacteria adhesion.* S. aureus*, known for its extensive resistance to antibiotics, is the most common cause of implant infections [3] and was thus chosen for the study. Bacteria can use molecular features of their cell membrane as sensors and are much less deformable than eukaryotic cells. Compared with conventional Ti, TNTs without antibiotics in the study reduced bacterial attachment. Our result is in contradiction to the report that increased bacteria attachment was observed on TNTs [18]. This difference in bacterial behavior compared with our data might be caused by the substantially different nature of the nanotubes (as-anodized, amorphous phase TNTs in their study versus heat-treated and crystallized, anatase phase TNTs in ours). TNTs were synthesized on the Ti surface by anodization in an electrolyte containing fluorine, which could remain on the surface of the nanotubes. However, fluorine has been reported to increase bacterial adhesion [18, 34, 35]. Our heat treatment, which allowed evaporating the residual fluorine, could reduce bacterial adhesion. Simultaneously, we found that the number of bacteria attached to the surface of TNTs surfaces decreased as the diameter increased. This finding is just opposite to the report that small diameter (20 nm) nanotubes produced more robust antimicrobial effect than big diameter (80 nm) nanotubes [15]. The difference in bacteria growth can be explained by the difference in the surface wettability. The hydrophilicity of the TNTs decreased with ascending diameter in their study, while it increased with ascending diameter in ours. Small diameter nanotubes in their study were rutile crystalline phase while all three different nanotubes in our study were anatase phase. Anatase is the preferred crystalline modification of titania as it provides a larger surface area compared with rutile [36–38], which could lead to the hydrophobicity of small diameter nanotubes in their study. Although it has been shown that both anatase and rutile phases possess antibacterial properties [39], underlying differences between the two possibly influence bacterial responses. Considering the common adhesion mechanism of various bacteria on implant surfaces [40, 41], we speculate that the results observed in* S. aureus* may translate to other species of bacteria as well.

## 5. Conclusions

In summary, TNTs with three different diameters were fabricated on Ti surface using weakly acidic electrolyte. Our results showed that NT30 improved BMSCs osteogenic differentiation to the utmost without blocking cell proliferation and simultaneously decreased* S. aureus* adhesion. Moreover, the fabrication process of TNTs is simple, economical, and easily repeatable. Thus, NT30 is highly attractive for biomedical implants because of its promotion of osseointegration and antibacterial efficacy without the use of pharmaceuticals.

## Figures and Tables

**Table 1:** Primers used for qRT-PCR.

Gene	Forward primer sequence (5′-3′)	Reverse primer sequence (5′-3′)
RUNX2	CCTCTGACTTCTGCCTCTGG	GATGAAATGCCTGGGAACTG
ALP	GCCTGGACCTCATCAGCATT	AGGGAAGGGTCAGTCAGGTT
OCN	CAAGTCCCACACAGCAACTC	CCAGGTCAGAGAGGCAGAAT
COL-1	ATCTCCTGGTGCTGATGGAC	GCCTCTTTCTCCTCTCTGACC
GAPDH	GGCACAGTCAAGGCTGAGAATG	ATGGTGGTGAAGACGCCAGTA

## References

[1] Actis L., Gaviria L., Guda T., Ong J. L. (2013). Antimicrobial surfaces for craniofacial implants: state of the art. *Journal of the Korean Association of Oral and Maxillofacial Surgeons*.

[2] Darouiche R. O. (2004). Treatment of infections associated with surgical implants. *The New England Journal of Medicine*.

[3] Campoccia D., Montanaro L., Arciola C. R. (2006). The significance of infection related to orthopedic devices and issues of antibiotic resistance. *Biomaterials*.

[4] Neoh K. G., Hu X., Zheng D., Kang E. T. (2012). Balancing osteoblast functions and bacterial adhesion on functionalized titanium surfaces. *Biomaterials*.

[5] Chua P.-H., Neoh K.-G., Kang E.-T., Wang W. (2008). Surface functionalization of titanium with hyaluronic acid/chitosan polyelectrolyte multilayers and RGD for promoting osteoblast functions and inhibiting bacterial adhesion. *Biomaterials*.

[6] Hu X., Neoh K.-G., Shi Z., Kang E.-T., Poh C., Wang W. (2010). An in vitro assessment of titanium functionalized with polysaccharides conjugated with vascular endothelial growth factor for enhanced osseointegration and inhibition of bacterial adhesion. *Biomaterials*.

[7] Johnson R. (2014). Antibiotics: resistance is restored. *Nature Chemistry*.

[8] Mendonça G., Mendonça D. B. S., Simões L. G. P. (2009). The effects of implant surface nanoscale features on osteoblast-specific gene expression. *Biomaterials*.

[9] Park J., Bauer S., von der Mark K., Schmuki P. (2007). Nanosize and vitality: TiO_2_ nanotube diameter directs cell fate. *Nano Letters*.

[10] Park J., Bauer S., Schlegel K. A., Neukam F. W., Von Mark K. D., Schmuki P. (2009). TiO_2_ nanotube surfaces: 15 nm—an optimal length scale of surface topography for cell adhesion and differentiation. *Small*.

[11] Oh S., Brammer K. S., Li Y. S. J. (2009). Stem cell fate dictated solely by altered nanotube dimension. *Proceedings of the National Academy of Sciences of the United States of America*.

[12] Brammer K. S., Oh S., Cobb C. J., Bjursten L. M., Heyde H. V. D., Jin S. (2009). Improved bone-forming functionality on diameter-controlled TiO_2_ nanotube surface. *Acta Biomaterialia*.

[13] Zhao L., Liu L., Wu Z., Zhang Y., Chu P. K. (2012). Effects of micropitted/nanotubular titania topographies on bone mesenchymal stem cell osteogenic differentiation. *Biomaterials*.

[14] Park M. R., Banks M. K., Applegate B., Webster T. J. (2008). Influence of nanophase titania topography on bacterial attachment and metabolism. *International Journal of Nanomedicine*.

[15] Kummer K. M., Taylor E. N., Durmas N. G., Tarquinio K. M., Ercan B., Webster T. J. (2013). Effects of different sterilization techniques and varying anodized TiO_2_ nanotube dimensions on bacteria growth. *Journal of Biomedical Materials Research Part B: Applied Biomaterials*.

[16] Ercan B., Taylor E., Alpaslan E., Webster T. J. (2011). Diameter of titanium nanotubes influences anti-bacterial efficacy. *Nanotechnology*.

[17] Ercan B., Kummer K. M., Tarquinio K. M., Webster T. J. (2011). Decreased *Staphylococcus aureus* biofilm growth on anodized nanotubular titanium and the effect of electrical stimulation. *Acta Biomaterialia*.

[18] Puckett S. D., Taylor E., Raimondo T., Webster T. J. (2010). The relationship between the nanostructure of titanium surfaces and bacterial attachment. *Biomaterials*.

[19] Yu W.-Q., Jiang X.-Q., Xu L., Zhao Y.-F., Zhang F.-Q., Cao X. (2011). Osteogenic gene expression of canine bone marrow stromal cell and bacterial adhesion on titanium with different nanotubes. *Journal of Biomedical Materials Research. Part B. Applied Biomaterials*.

[20] Cipriano A. F., Miller C., Liu H. (2014). Anodic growth and biomedical applications of TiO_2_ nanotubes. *Journal of Biomedical Nanotechnology*.

[21] Lord M. S., Foss M., Besenbacher F. (2010). Influence of nanoscale surface topography on protein adsorption and cellular response. *Nano Today*.

[22] Grinnell F., Feld M. K. (1982). Fibronectin adsorption on hydrophilic and hydrophobic surfaces detected by antibody binding and analyzed during cell adhesion in serum-containing medium. *The Journal of Biological Chemistry*.

[23] Webb K., Hlady V., Tresco P. A. (1998). Relative importance of surface wettability and charged functional groups on NIH 3T3 fibroblast attachment, spreading, and cytoskeletal organization. *Journal of Biomedical Materials Research*.

[24] Borges A. M. G., Benetoli L. O., Licínio M. A. (2013). Polymer films with surfaces unmodified and modified by non-thermal plasma as new substrates for cell adhesion. *Materials Science and Engineering C*.

[25] Wang Y., Wen C., Hodgson P., Li Y. (2014). Biocompatibility of TiO_2_ nanotubes with different topographies. *Journal of Biomedical Materials Research Part A*.

[26] Liu X., Feng Q., Bachhuka A., Vasilev K. (2013). Surface chemical functionalities affect the behavior of human adipose-derived stem cells in vitro. *Applied Surface Science*.

[27] McBeath R., Pirone D. M., Nelson C. M., Bhadriraju K., Chen C. S. (2004). Cell shape, cytoskeletal tension, and RhoA regulate stem cell lineage commitment. *Developmental Cell*.

[28] Kilian K. A., Bugarija B., Lahn B. T., Mrksich M. (2010). Geometric cues for directing the differentiation of mesenchymal stem cells. *Proceedings of the National Academy of Sciences of the United States of America*.

[29] Stein G. S., Lian J. B., Owen T. A. (1990). Relationship of cell growth to the regulation of tissue-specific gene expression during osteoblast differentiation. *The FASEB Journal*.

[30] Wall I., Donos N., Carlqvist K., Jones F., Brett P. (2009). Modified titanium surfaces promote accelerated osteogenic differentiation of mesenchymal stromal cells in vitro. *Bone*.

[31] Cavalcanti-Adam E. A., Aydin D., Hirschfeld-Warneken V. C., Spatz J. P. (2008). Cell adhesion and response to synthetic nanopatterned environments by steering receptor clustering and spatial location. *HFSP Journal*.

[32] Fux C. A., Costerton J. W., Stewart P. S., Stoodley P. (2005). Survival strategies of infectious biofilms. *Trends in Microbiology*.

[33] Gottlieb T., Atkins B. L., Shaw D. R. (2002). 7: soft tissue, bone and joint infections. *The Medical Journal of Australia*.

[34] Katsikogianni M., Spiliopoulou I., Dowling D. P., Missirlis Y. F. (2006). Adhesion of slime producing *Staphylococcus epidermidis* strains to PVC and diamond-like carbon/silver/fluorinated coatings. *Journal of Materials Science: Materials in Medicine*.

[35] Li B., Logan B. E. (2004). Bacterial adhesion to glass and metal-oxide surfaces. *Colloids and Surfaces B: Biointerfaces*.

[36] Liu B., Khare A., Aydil E. S. (2012). Synthesis of single-crystalline anatase nanorods and nanoflakes on transparent conducting substrates. *Chemical Communications*.

[37] Park J. T., Patel R., Jeon H., Kim D. J., Shin J.-S., Kim J. H. (2012). Facile fabrication of vertically aligned TiO_2_ nanorods with high density and rutile/anatase phases on transparent conducting glasses: high efficiency dye-sensitized solar cells. *Journal of Materials Chemistry*.

[38] Lee K., Kim D., Roy P. (2010). Anodic formation of thick anatase TiO_2_ mesosponge layers for high-efficiency photocatalysis. *Journal of the American Chemical Society*.

[39] Del Curto B., Brunella M. F., Giordano C. (2005). Decreased bacterial adhesion to surface-treated titanium. *The International Journal of Artificial Organs*.

[40] Anselme K., Davidson P., Popa A. M., Giazzon M., Liley M., Ploux L. (2010). The interaction of cells and bacteria with surfaces structured at the nanometre scale. *Acta Biomaterialia*.

[41] Peng Z., Ni J., Zheng K. (2013). Dual effects and mechanism of TiO_2_ nanotube arrays in reducing bacterial colonization and enhancing C3H10T1/2 cell adhesion. *International Journal of Nanomedicine*.

